# Gamma Knife Surgery as Monotherapy with Clinically Relevant Doses Prolongs Survival in a Human GBM Xenograft Model

**DOI:** 10.1155/2013/139674

**Published:** 2013-11-10

**Authors:** Bente Sandvei Skeie, Jian Wang, Ernest Dodoo, Jan Ingeman Heggdal, Janne Grønli, Linda Sleire, Sidsel Bragstad, Jeremy C. Ganz, Martha Chekenya, Sverre Mørk, Paal-Henning Pedersen, Per Øyvind Enger

**Affiliations:** ^1^Department of Neurosurgery, Haukeland University Hospital, 5021 Bergen, Norway; ^2^Institute of Surgical Sciences, Haukeland University Hospital, 5021 Bergen, Norway; ^3^Oncomatrix Research Lab, Department of Biomedicine, University of Bergen, 5021 Bergen, Norway; ^4^Department of Neurosurgery, Karolinska University Hospital, 171 76 Stockholm, Sweden; ^5^Department of Oncology and Medical Physics, Haukeland University Hospital, 5021 Bergen, Norway; ^6^Department of Biological and Medical Psychology, University of Bergen, 5021 Bergen, Norway; ^7^Norwegian Competence Center for Sleep Disorders, Haukeland University Hospital, 5021 Bergen, Norway; ^8^Brain Tumor Immunology & Therapy Group, Department of Biomedicine, University of Bergen, 5021 Bergen, Norway

## Abstract

*Object*. Gamma knife surgery (GKS) may be used for recurring glioblastomas (GBMs). However, patients have then usually undergone multimodal treatment, which makes it difficult to specifically validate GKS independent of established treatments. Thus, we developed an experimental brain tumor model to assess the efficacy and radiotoxicity associated with GKS. *Methods*. GBM xenografts were implanted intracerebrally in nude rats, and engraftment was confirmed with MRI. The rats were allocated to GKS, with margin doses of 12 Gy or 18 Gy, or to no treatment. Survival time was recorded, tumor sections were examined, and radiotoxicity was evaluated in a behavioral open field test. *Results*. In the first series, survival from the time of implantation was 96 days in treated rats and 72 days in controls (*P* < 0.001). In a second experiment, survival was 72 days in the treatment group versus 54 days in controls (*P* < 0.006). Polynuclear macrophages and fibrosis was seen in groups subjected to GKS. Untreated rats with GBM xenografts displayed less mobility than GKS-treated animals in the open field test 4 weeks after treatment (*P* = 0.04). *Conclusion*. GKS administered with clinically relevant doses prolongs survival in rats harboring GBM xenografts, and the associated toxicity is mild.

## 1. Introduction

The current treatments for GBMs include surgery, fractionated radiotherapy (FR), and temozolomide. This approach provides a definite effect as patients receiving this multimodal treatment have a median survival of approximately 15 months [1, 2], compared with 3 months if no treatment is given. Surgical debulking reduces symptoms and provides tissue for diagnosis, but infiltrative tumor growth makes complete removal impossible. Conventional radiotherapy improves survival [3] but is associated with noteworthy toxicity due to the high doses delivered to the surrounding brain tissue. Thus, patients surviving more than 12 months often exhibit significant cognitive deficits. Due to the improved survival of GBM patients in recent years with two year survival rates of 26.5%, more people will live to experience these side effects [4]. As such, optimized radiation modalities to reduce toxicity are warranted. Future treatments may include lower dose FR combined with hypofractionated stereotactic radiosurgery and additional tumor selective radiosensitizers. To explore innovative regimens for treating malignant glioma, a representative animal model is needed.

GKS enables the delivery of a high tumor dose in one single fraction by converging multiple beams to a stereotactically defined target, creating a sharp dose fall-off at the tumor margin with minimal radiation to the surrounding brain [5]. GKS is not used in the primary treatment of GBMs, but there are several reports describing its use for GBM recurrences in patients that have already received primary treatment, including FR [6–9]. Thus, little is known about the effects specifically associated with GKS.

However, most GBM recurrences are local, suggesting a role for focused radiation [10]. Moreover, experimental studies suggest a therapeutic benefit with focused radiation of rats implanted with rat C6 and rat 9L glioma cells [11, 12]. These rat glioma cell lines however, do not mimic the infiltrative growth of human GBMs. Furthermore, previous animal studies have involved treatment with lateral and posterior-anterior plain X-rays [11] or additional therapies such as injection of viral vectors [12]. Thus, there remains a need to evaluate the radiobiology of GKS with clinically relevant doses in tumor models that mimic the infiltrative growth of human GBMs. We previously established highly infiltrative brain tumors in nude rats from human GBM biopsies [14]. Direct xenografting of patient GBM biopsies into nude rats has been shown to produce tumors with infiltrative growth patterns without angiogenesis, similar to low-grade glioma [13]. Serial passaging of these tumors in vivo transforms purely invasive phenotypes into mature GBM phenotypes that are also angiogenesis-dependent and necrotic [13, 14]. In this study, we used this highly representative GBM model to evaluate the effect of GKS treatment on survival, local tumor control, and radiotoxicity.

## 2. Material and Methods

### 2.1. Tumor Spheroids

GBM biopsies were obtained during surgical resections performed at the Department of Neurosurgery at Haukeland University Hospital (Bergen, Norway), with informed consent from the patients and approval by the regional ethical committee. The biopsy spheroids were prepared as described previously [15] and passaged for several generations in immunodeficient rats [13]. Tumor spheroids were prepared from two GBM patients, pA and pB.

### 2.2. Animal Experiments

In total, 39 male and female athymic, homozygous nude rats (rnu/rnu, Rowett) between 200 and 250 g were used in 3 different experiments (Table 1). They were kept on a standard pellet diet, in a pathogen free environment at a constant temperature and humidity, on a standard 12/12 h light and dark cycle. The animals were provided a standard pellet diet and tap water *ad libitum*. All experimental procedures were approved by the Norwegian Animal Research Authority (Oslo, Norway). Prior to implantation, all animals were anesthetized with Isoflurane gas (1.5% mixed with 50% air and 50% O_2_) and 0.5% Marcaine subcutaneously. Intracranial tumor implantations were conducted with a stereotactic frame (Kopf instruments, Tujunga, CA, USA) as previously described [14].

In the first experiment, animals with comparable tumor volumes derived from a patient GBM xenograft, pA, were randomly assigned to 3 different groups: (1) GKS with 12 Gy margin dose; (2) GKS with 18 Gy margin dose; and (3) untreated controls. In the second experiment, we used another GBM xenograft, pB, and the animals were randomized to two groups: (1) GKS with 12 Gy margin dose and (2) untreated controls. Finally, 6 animals were grafted with pA tumor material in a separate experiment to assess radiotoxicity (see Section 2.6 describing the open field study). Animals were inspected daily by the staff at the animal facility that were blinded to the treatments the animals had undergone. Animals were sacrificed with CO_2_ at the onset of symptoms such as weight loss, neurological deficits, passiveness, or other signs of illness. All animals were observed until the onset of symptoms or until 165 days after implantation. The brains were removed and fixed in 4% formalin. Survival was used as primary endpoint and histological changes and volume changes on MRI follow-up were used as secondary endpoints.

### 2.3. Magnetic Resonance Imaging

Animals implanted with pA and pB tumor spheroids underwent MRI scanning 6 and 7 weeks after implantation, respectively, using a Bruker Pharmascan 7 T small animal MRI (Bruker Biospin MRI GmnH, Ettingen, Germany). Animals implanted with pB GBM spheroids in the second experiment also underwent a repeated scanning one week later. Axial T1 and T2 weighted images were acquired as previously described [14]. The tumor volumes at treatment and on follow-up MRI were measured in Gamma Plan (Elekta Instrument AB, Stockholm, Sweden).

### 2.4. Gamma Knife Treatment

**Figure 1:**
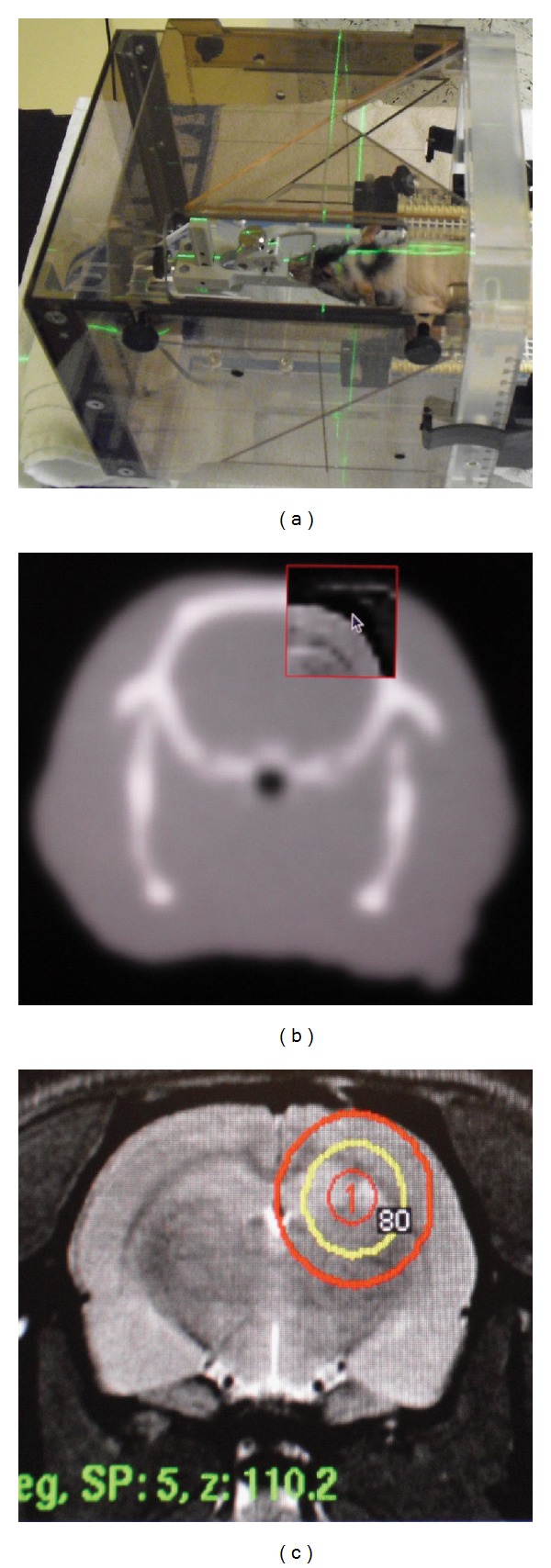
GKS of nude rats harboring GBM xenografts. The rats are fixed in a stereotactic frame (a) attached to a transparent hood prior CT scanning (b). These scans are merged with MRI images (c). Shown is a dose administered with a 4 mm collimator (red) and the accompanying 80% isodose curve (yellow).

Animals were irradiated with the Leksell Gamma Knife Perfexion (Elekta Instrument AB, Stockholm, Sweden) anesthetized with intramuscular Hypnorm-Dormicum (0, 4 mL/kg). One day after MRI, the rats were immobilized in a Regis-Valliccioni stereotactic frame 5 (Neuropace, Neuilly, France (Figure 1(a))) [16] and underwent CT scanning with the frame attached. The MRI images of the tumors were coaligned with the CT scans in the Gammaplan, aided by anatomical landmarks and the visible trajectory from the tumor implantation (Figure 1(b)). The rats were randomized to different treatment groups and treated with a tumor margin dose of 12 Gy or 18 Gy to the 50–88% (mean 74.6%) isodose, using collimator size 4 (Figure 1(c)), or they were randomized to no treatment. The mean treatment time was 5.0 minutes. In the radiotoxicity experiment, the rats received early GKS only 9 days after implantation to allow for a long follow-up time. These animals received 12 Gy to the 80% isodose, centered at the site of the implantation (Figure 1(c)).

### 2.5. Histology and Immunohistochemical Analyses

Tissue blocks were paraffin embedded and 5 *μ*m sections were obtained. These were stained with haematoxylin and eosin and examined by a neuropathologist. Sections were prepared as described previously [13] and immunostained for Nestin (1 : 1000, Chemicon, Temecula, CA, USA), Vimentin (1 : 500, DAKO, Glostrup, Denmark), and Ki-67 (MIB-1, 1 : 500, Dako). Cell death was determined using the terminal transferase-mediated dUTP nick-end-labeling (TUNEL) method according to the manufacturer's instructions (Boehringer Mannheim, Mannheim, Germany) using DAB as a chromogen. TUNEL staining was quantitated as % immunopositive area fraction at 400x magnification, using a Nikon light microscope (THP Eclipse E600, Nikon Corporation, Tokyo, Japan) and the NIS-Elements BR 4.0 software (Nikon Corporation). Four hotspot fields of view were analyzed per animal from each treatment group and control group and quantified by a person blinded to the groups.

### 2.6. Open Field Experiment

In order to evaluate locomotor and exploratory behavior deficits as a measure of radiotoxicity, we performed an open field test 2 days and 4 weeks after randomization to treatment or no treatment: (1) GKS after tumor implantation (3 animals); (2) no treatment after tumor implantation (3 animals); and (3) controls with no tumor nor treatment (3 animals). The open field behavioral test was developed by Calvin S. Hall [17], and measures general locomotor activity and willingness to explore in rodents. The field consists of a square black box, 1 m × 1 m with 40 cm high walls. All animals were placed in the centre of the open field arena and the animals' spontaneous activities for 9 minutes were digitally recorded. Latency to enter the periphery (indicative of anxiety) and central zone, angular velocity, which is the animals' ability to turn in a specific direction, general mobility, and time spent in left or right part of the box were measured.

### 2.7. Statistical Methods

Animal survival was registered from the time of implantation and from the time of treatment. Survival was plotted on a Kaplan-Meyer survival curve, and median survival times were compared using the log-rank test in SPSS version 18.0. Univariate analyses of categorical variables according to treatment group (12 Gy versus 18 Gy), 12 Gy volume outside the tumor (<30 mm³ versus >70 mm³), maximum dose (<20 Gy versus >20 Gy), tumor volume (<5 mm³ versus 5–15 mm³ versus >15 mm³), and the percentage isodose used during treatment (<80% isodose curve versus >80% isodose curve) were conducted. Tumor volumes on T1 weighted MRI images on the day of the treatment and follow-up images were measured based on the contrast-enhancing areas to detect differences in tumor volumes, and the treatment groups were compared using Student's *t*-test. Ki-67 labeling and apoptotic indices were analyzed using ANOVA. The open field experiments were analyzed using ANOVA for repeated measures and the post hoc Fisher LSD test. A probability value ≤0.05 was regarded significant.

## 3. Results

### 3.1. Survival

**Figure 2:**
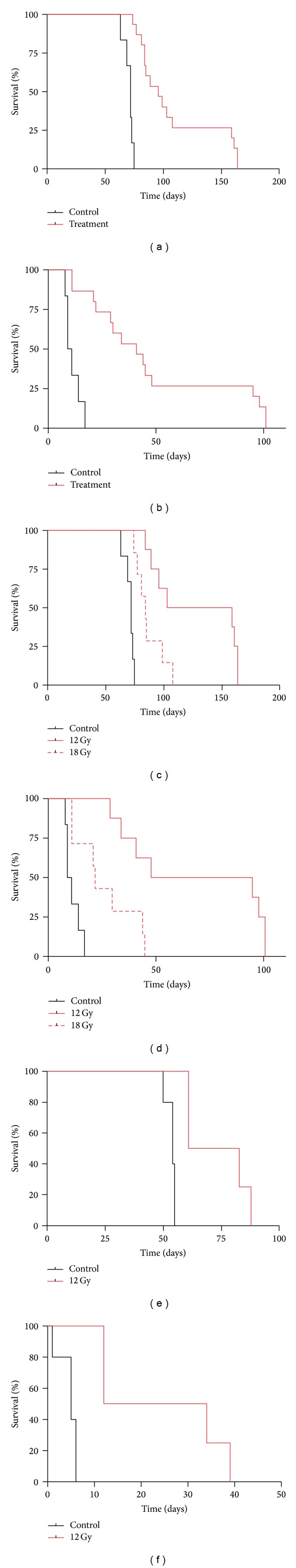
GKS prolongs survival in nude rats harboring GBM xenografts. Shown are the treatment groups as indicated for the experiment using pA (a–d) and pB (e) and (f) GBM xenografts. Treated rats (both 12 and 18 Gy collectively) versus controls from the time of implantation, (a) and from the time of treatment (b). Comparison of the individual treatment groups from the time of implantation (c) and from the time of treatment (d). Survival from the time of tumor (pB) implantation (e) and from the time of treatment (f).

In the first experiment using pA GBM xenografts, 8 rats received a 12 Gy margin dose (mean volume 12.0 ± 7.9 mm³), 7 rats received an 18 Gy margin dose (mean volume 8.6 ± 5.9 mm³), and 6 rats served as controls (mean volume 10.0 ± 6.7 mm³, *P* < 0.65). The median survival from the time of implantation was 96 days (95% CI 78.3–113.7) for the two treatment groups collectively, compared to 72 days (95% CI 68.6–75.4) for the untreated rats (*P* < 0.0001, Figure 2(a)). The median survival from the time of GKS was 41 days (95% CI 23.3–58.6) for the GKS treated rats compared to 10 days (95% CI 6.6–11.4) for the untreated rats (*P* < 0.0001, Figure 2(b)). Notably, the median survival time both from implantation and from the time of treatment was significantly longer for animals treated with 12 Gy (131 days from implantation, 72 days from GKS), compared to animals treated with a 18 Gy margin dose (84 days and 22 days, respectively, *P* < 0.001, Figures 2(c) and 2(d).

The rats treated with an 18 Gy margin dose had a mean maximum dose of 23.0 ± 1.7 Gy, whereas the rats treated with a 12 Gy margin dose had a mean maximum dose of 15.5 ± 1.9 Gy (*P* < 0.001). Furthermore, for the rats receiving 18 Gy the mean 12 Gy volume outside the tumor was 77.9 ± 10.7 mm³ versus 20.7 ± 7.7 mm³ for the rats receiving 12 Gy (*P* < 0.001). A multivariate analysis according to margin dose and adjusted for maximum dose, tumor volume, and volume of normal brain receiving >12 Gy confirmed a significantly longer survival in the 12 Gy group (*P* < 0.02).

In the second series, spheroids from the pB GBM xenograft produced a more rapid disease course than the pA tumors (Figures 2(e) and 2(f)). At the time of GKS 7 weeks after implantation, the tumors had reached a bigger volume than in the first series. The tumors were treated with 12 Gy to the periphery. The mean volume for the pB tumors was 120 mm³ (versus 10.3 mm³ for the pA tumors), with no significant differences between the 4 treated animals and the 5 controls. However, even for these late stage tumors, median survival was significantly longer for the GKS treatment group both from the time of implantation, 72 days versus 54 days (*P* = 0.006, Figure 2(e)), and from GKS, 12 days versus 5 days (*P* = 0.006, Figure 2(f)).

### 3.2. MRI Monitoring of Tumor Growth

**Figure 3:**
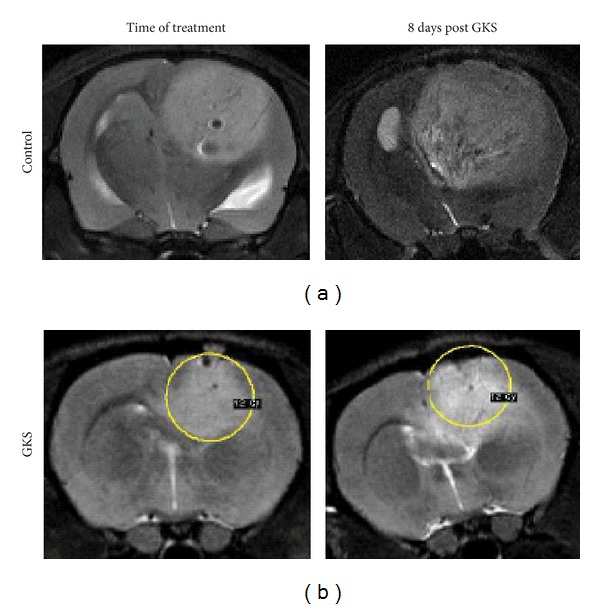
MRI imaging demonstrates reduced tumor growth following GKS treatment. Shown are T2-sequences, with tumor lesion presenting as a high signal intensity area, compared to the surrounding brain. Untreated rat brain tumor (left) and tumor receiving 12 Gy margin dose (right) at the day of treatment (a). Untreated rat brain tumor (left) and GKS treated tumors (right) one week after treatment (b). The yellow line indicates the location of the 12 Gy dose at the time of treatment.

In the second experiment, with pB tumors, MRI performed 8 days after the initial MRI showed a larger increase in tumor volume for the untreated controls compared to rats subjected to GKS (Figure 3). The mean difference in tumor volumes between baseline and follow-up was 111.8 mm³ for the 12 Gy group versus 252.8 mm³ for the controls (*P* = 0.032). The tumors increased in volume by a factor of 2.9 for the controls versus a factor of 2.1 for the treated rats.

### 3.3. Tumor Histology

**Figure 4:**
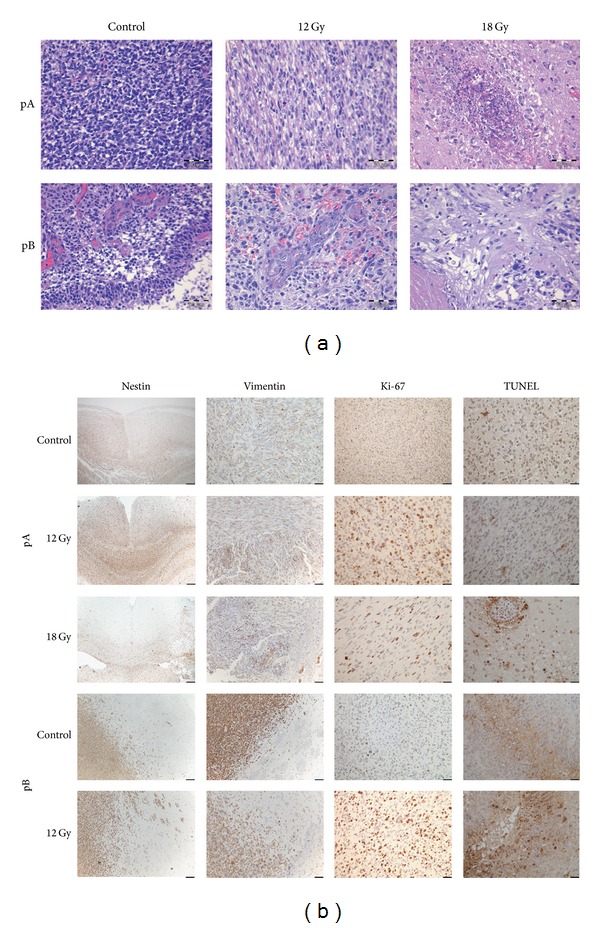
H/E and IHC staining of GBM xenografts. H/E staining of the tumors (a) which appear hypercellular, but with more prominent angiogenesis and necrosis in the pB GBM xenografts (lower panels). Granulomatous cerebral angiitis with polynuclear macrophages in the perivascular space can be seen in the pA GBM xenograft treated with 18 Gy (Upper right panel). Scale bars: 50 *μ*m. IHC and TUNEL staining (brown) show robust expression of Nestin, Vimentin, and Ki-67 and TUNEL positive cells. Panels at low magnification show Nestin positive cells in the pA xenograft migrating along the Corpus Callosum (b). Scale bars pA: Nestin 250 *μ*m, Vimentin: 100 *μ*m, Ki-67 from pA ctrl: 100 *μ*m, all other panels: 25 *μ*m. Scale bars pB: Nestin and Vimentin: 100 *μ*m, all other panels: 50 *μ*m.

Both the pA and pB GBM xenografts produced tumors in all rats that were implanted. The pA tumors showed predominantly infiltrative growth with extensive cellular infiltration throughout the brain, whereas the pB tumors predominantly displayed microvascular proliferation with necrosis (Figures 4(a) and 4(b)). Brains from the treatment groups also showed histological changes suggesting granulomatous cerebral angiitis with polynuclear macrophages in the perivascular space, tumor cells in the Virchow-Robin space, and fibrosis (Figure 4(a), upper right panel). Notably, the number of microscopic fields (×10) with the presence of granulomatous angiitis were significantly higher in the group receiving 18 Gy (49%) compared to the group receiving 12 Gy (8%, *P* = 0.02).

Recently, several studies have suggested that cancer stem cells mediate radioresistance in gliomas [18–20]. Thus, we investigated the expression of stem cell markers Nestin and Vimentin (Figure 4(b)). We observed Nestin and Vimentin expression in the treatment groups as well as the controls, both in the tumor bulk and the invasion zone. Labeling index for the proliferation marker Ki-67 did not differ significantly between any of the groups. In the first experiment, control tumors displayed 18 ± 2.0% Ki-67 positive cells, tumors treated with 12 Gy contained 19 ± 3.9% positive cells, whereas 18 Gy treated tumors had 18 ± 0.7% Ki-67 positive cells (*P* = 0.99). Apoptosis estimated by % area fractions of TUNEL-positive staining did not show an increase with radiation dose and were 0.87% for the controls, 0.204 for the 12 Gy group, and 2.210 for the 18 Gy group, (Figure 4(b), right panels).

### 3.4. Open Field Test

**Figure 5:**
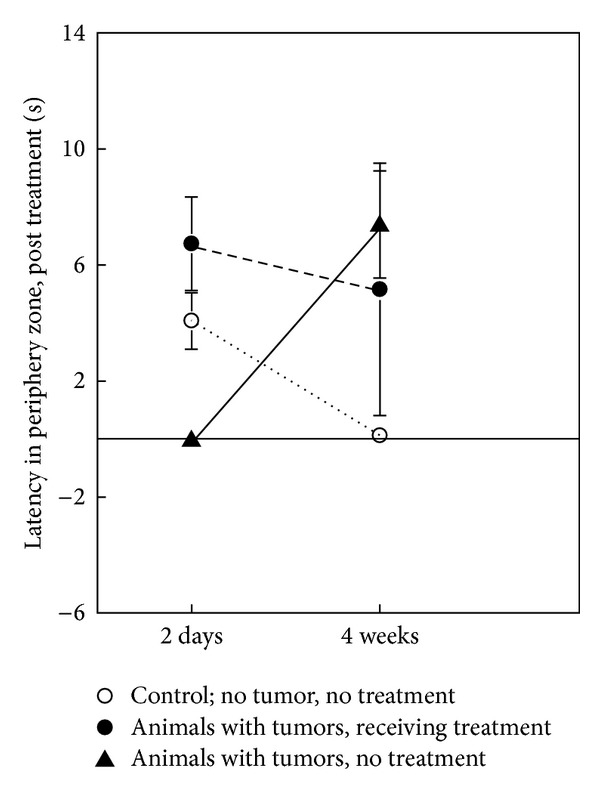
Behavioral open field study comparing rats harboring GBM xenografts with and without GKS treatment, as well as controls. Graphic presentation of latencies to enter the periphery for nude rats, 2 days and 4 weeks after treatment. Three animals were tested from each group as indicated.

In order to evaluate radiotoxic side effects, we performed an open field test two days and four weeks following treatment (Figure 5). We compared the activity and exploratory behavior of rats with GBM receiving GKS with the behavior of both untreated rats harboring GBM tumors and untreated rats without tumors (controls). Two days after GKS, the treated rats had a longer latency to enter the periphery zone (*P* = 0.005) and seemed less active in the open field (*P* = 0.09) than untreated rats with tumors. After 4 weeks, all rats with tumors were less mobile than the controls. Notably, the latency for moving to the outer ring was longer in the untreated rats with tumors than the treated rats with brain tumors (*P* = 0.04) and the controls with no tumor (*P* = 0.002).

## 4. Discussion

The potential for GSK in GBM management has so far not been fully elucidated, whereas the Stupp regime is widely adapted as a first line of treatment. However, GSK can be useful for small, deep seated, or recurrent tumors. Herein we assessed the efficacy of GSK treatment and its potential toxicity in a relevant model of GBM in nude rats. We established that GSK treatment prolonged survival of animals and that this was associated with minimal toxicity as indicated by mild histological and behavioral changes.

The accuracy of the Regis-Valliccioni frame has been documented previously [21, 22]. Moreover, the precision of 12 and 18 Gy dose delivery in our study was verified firstly by the presence of radiation-induced changes in the isodose center on histological examination in the rats receiving GKS and secondly by reduced tumor growth on follow-up MRI.

Previously, Kondziolka et al. reported prolonged survival after GKS for C6 glioma in rats receiving margin doses in the range of 30–100 Gy [11], whereas the survival seemed independent of the dose given. Khil et al. found a dose-dependent improvement in survival using margin doses of 25 Gy, 35 Gy, and 44 Gy in rats with 9L glioma cells [23]. However, these experiments included non-human, chemically induced glioma cell lines treated with high doses which cannot be administered clinically in single fraction stereotactic radiosurgery to previously irradiated patients. Accordingly, there remains a need to evaluate the radiobiological effects of GKS with doses relevant to the clinical setting of tumor relapse in a patient that has previously received FR.

In this study, GBM xenografts derived from patient tumors displayed the typical morphology of human GBMs, including infiltrative growth, florid angiogenesis, and necrotic regions. Moreover, the tumors were visible on MRI at the time of treatment, resembling the treatment setting in patients. In clinical studies validating the use of GKS for tumor recurrences in GBM patients, the doses have varied between 12 and 18 Gy [6–8]. Therefore, we selected 12 Gy and 18 Gy as representative clinical doses for our experiments. Our data demonstrate that monotherapy with GKS using clinically relevant doses prolongs survival. With a similar efficacy in patients, GKS may be an alternative to surgery in cases with deep seated small GBMs, when a diagnostic biopsy has been performed in patients who are otherwise unable to undergo a full craniotomy under narcosis due to severe comorbidity. Although most GBMs are too large for GKS at the time of diagnosis, GKS could be preferable to conventional radiotherapy in the initial treatment of small tumors due to a sharper dose fall and less toxicity [24]. Moreover, GKS in combination with lower, less toxic doses of conventional radiotherapy may improve future results.

Interestingly, rats treated with 12 Gy to the tumor margin lived significantly longer survival than rats treated with an 18 Gy margin dose. The reduced survival in the 18 Gy group could be caused by edema and radiation damage to adjacent brain tissue as a considerable volume of normal brain received more than 12 Gy in this group, which is seen as the limit for radiation damage of the normal human brain [25]. Polynuclear macrophage clusters in the perivascular space and fibrosis as seen in radiation induced granulomatous cerebral angiitis were found in the 18 Gy rats. Moreover, increased radiation injury with the 18 Gy treatments may trigger immune-mediated damage of healthy nervous tissue. However, it should be pointed out that even the 4 mm collimator becomes a crude tool for dose delivery, given the small rat brain volumes. Therefore, the extratumoral 12 Gy volumes in these animals are relatively much larger than in patients, and the observed toxicity may therefore have a very limited relevance to the clinical setting. In open field studies, animals with untreated tumors, animals treated with 12 Gy, and controls were tested. The rats treated with 12 Gy fared better than rats with untreated tumors. In addition, rats treated with 12 Gy improved from 2 days to 4 weeks after GKS, showing a shorter latency for moving to the outer ring of the open field arena after the start of the test. The data indicate a positive long-lasting effect of the treatment on rats' locomotor behavior. These animals readily started moving around, whereas animals receiving no treatment responded with more immobility or freezing in the start of the test. The latency to the outer zone showed large variation between individuals; however, the effects of the treatment on this parameter were robust compared to animals with untreated tumors. Thus, GKS using the 12 Gy dose seemed to have low toxicity with little impact on behavior. Notably, controls without tumor and no treatment also performed significantly better than untreated rats with tumors. Thus, the impact of the location of tumors on the locomotor behavior of the animal is likely as important as GKS.

## 5. Conclusion

We conclude that GKS administered as monotherapy with clinically relevant doses prolongs survival with low associated toxicity in rats harboring GBM xenografts. This in vivo nude rat model displays tumor implants highly representative of human GBMs. Moreover, the precision of dose delivery was verified by reduced tumor growth and the presence of radiation-induced histological changes in the rats subjected to GKS. The model described represents a valuable tool for future small animal studies. Additional studies on potential drug modification of the radiosurgery response in human GBM are warranted.

## Figures and Tables

**Table 1:** Overview with characteristics of the animal experiments conducted.

Experiment	Biopsy	Design	Radiation dose	Animals No.
No. 1	pA	therapeutic	12 and 18 Gy	21
No. 2	pB	therapeutic	12 Gy	9
No. 3	pA	open field test	12 Gy	9
